# “Talking About Your Medications”: A workshop series aimed at helping older adults participate in conversations about their medications

**DOI:** 10.1177/17151635221076075

**Published:** 2022-02-14

**Authors:** Barbara Farrell, Daniel Dilliott, Lisa Richardson, James Conklin, Lisa M. McCarthy, Lalitha Raman-Wilms

**Affiliations:** Bruyère Research Institute, Ottawa, Ontario; Department of Family Medicine, University of Ottawa, Ottawa, Ontario; Bruyère Research Institute, Ottawa, Ontario; Bruyère Research Institute, Ottawa, Ontario; Bruyère Research Institute, Ottawa, Ontario; Concordia University, Montreal, Quebec; Bruyère Research Institute, Ottawa, Ontario; Leslie Dan Faculty of Pharmacy and Department of Family and Community Medicine; University of Toronto, Toronto, Ontario; and the College of Pharmacy; Rady Faculty of Health Sciences, University of Manitoba, Winnipeg, Manitoba

## Introduction

Over the last 8 years, our Bruyère Research Institute–led deprescribing research team has coordinated the development of deprescribing guidelines^1-5^ and knowledge mobilization tools^6,7^ and worked with health care providers on implementation strategies.^8-13^ Our goal has been to provide evidence-based information that helps clinicians make decisions about when and how to safely reduce or stop unnecessary or problematic medications. This is important because the use of multiple medications can lead to adverse drug reactions and interactions, complicated regimens that affect medication understanding and adherence and contribute to falls, cognitive impairment, functional decline, emergency room visits and hospitalizations.^14^ Globally, the focus on medication overload, polypharmacy and the need for strategies to address these problems has grown.^15-17^ Shared decision making, as a tool for clinicians to make decisions with their patients about medications, is increasingly important.^18^

When our work began, health care providers told us that they would be more inclined to have conversations about deprescribing with patients if the topic were raised by the patient. Through our own experiences and from the literature, we know that people are challenged by minimal knowledge about their medications and by concerns (even fears) of approaching their health care provider with questions.^19^ Using a community-based participatory research approach, we worked with a local advisory group to understand older people’s challenges with polypharmacy and medication use.^20^ Our intention was to develop a locally relevant intervention to help patients collaborate with health care providers when making medication decisions, including deprescribing. Our local advisory group recommended providing a series of small group, live-delivery, interactive workshops to help participants build knowledge about medication management and polypharmacy and to develop skills and confidence to participate in medication decisions.

We initially planned to deliver face-to-face workshops in the spring of 2020, but due to the pandemic we adapted the workshops for virtual delivery. To our knowledge, this is the first time that health care providers have delivered a series of interactive, discussion-based, online workshops about deprescribing, polypharmacy and medication management for older community-dwelling adults. This unique experience for our team highlighted the important role that pharmacists and other health care providers can play in engaging with people virtually to deliver education and enable them to participate in their own health care. This is especially important at a time when older people may feel isolated and want opportunities for social connectedness.

This article describes the development and piloting of these workshops and offers suggestions to others who may wish to use these materials.

## Workshop design and delivery

In designing the workshops, the team intended to use diverse teaching and learning strategies. Aligned with the principles of adult learning, we included active participation, reflection and simulation and availability of supplemental information to support self-directed learning (Table 1). In collaboration with the local advisory group, we developed learning objectives and content for 3 interactive, 90-minute workshops to be facilitated by a health care provider with knowledge of polypharmacy, deprescribing, medication management and shared decision making. The workshops were designed so that knowledge and skills are acquired in a progressive manner, from basic to more complex concepts and practices, to enable understanding of the importance of the participant’s active involvement in medication management decisions before learning specifically about deprescribing. To support attainment of each workshop’s learning objectives, the team drew from resources related to deprescribing, shared decision making and medication management.^18,21-27^ The team also created worksheets for interactive learning activities, homework assignments between workshops, a video illustrating a shared decision-making conversation between a patient and health care provider and additional resources to support attendees in applying their learning. An overview of the workshop series, with the learning objectives, is provided in Table 1.

**Table 1:** Overview of workshop series

Workshop	General description	Learning objectives	Teaching and learning strategies used throughout
Workshop 1: Talking About Your Medications	This session provides an introduction to the workshop series as well as an overview of polypharmacy and deprescribing. It also reviews the implications of polypharmacy and deprescribing and the benefits and outcomes from appropriate medication management.	Participants will be able to 1. Describe the problems of polypharmacy among older adults. 2. Indicate how problematic polypharmacy may be part of their own medication management experience. 3. Describe some of the benefits that can result from appropriate medication management. 4. Explain how deprescribing is part of good prescribing and ideal medication management.	**Active participation** • Posing questions designed to stimulate conversation and the sharing of personal experiences **Reflection** • Through homework activities **Simulation** • Observing conversations **Supplemental information** • Additional resources to convey and reinforce concepts
Workshop2: Getting Reliable Medication Information	This session has 3 parts: what participants need to know about their medications and why it is important, how to keep track of this information in a way that is organized and easy to use and where to find reliable medication resources if participants have questions.	Participants will be able to 1. Describe what they need to know about their medications and why it is important. 2. Keep track of their medication information in a way that is organized and easy to use. 3. Use reliable medication resources if they have questions.	
Workshop 3: Having Conversations About Your Medications With Your Health Care Providers	This session introduces the concepts of shared decision making and the circle of care and supports participant engagement with their health care providers in managing their medications.	Participants will be able to 1. Identify questions they have about their medications. 2. Explain the idea of shared decision making as part of useful medication conversations. 3. Play an important role in managing and making decisions about their medications.	

The virtual workshop series pilot took place over 3 weeks in November 2020. It was hosted in partnership with Living Healthy Champlain, which helped advertise, handled registration and provided access to its Zoom videoconferencing account.^28^ Eight people registered within hours of advertising; they were mailed Participant Workbooks (Appendix 1, available online atwww.cpjournal.ca) 1 week before the first workshop and emailed a link to the video conference the day before each workshop.

The workshops were facilitated by a pharmacist team member. The project coordinator also attended to provide technical support (e.g., ensure microphones and webcams were functioning properly, view the chat box for questions, support participants who had difficulty locating the materials in their Participant Workbook via chatbox). The facilitator delivered content using their camera only, ensuring continuous visual contact with participants, while they followed along with the PowerPoint slide content in their workbooks. The facilitator periodically paused while delivering content in order to engage the group in sharing their thoughts and feelings on a topic.

### Workshop implementation materials

**Figure 1:**
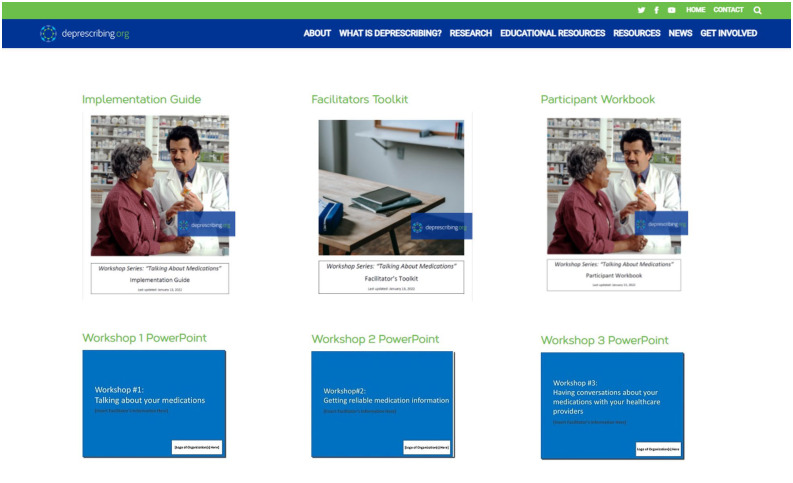
Overview of workshop materials made freely available on the development team’s website https://deprescribing.org/talking-about-medications-workshop-materials

Informed by this experience and feedback from attendees, our team developed an Implementation Guide and Facilitator’s Toolkit and revised the Participant Workbook, to enable easy use by other health care providers interested in hosting workshops in their own communities. The materials were reviewed by 6 external reviewers (pharmacists, pharmacy students, a local advisory group member) for usability and then posted on the research team’s website (https://deprescribing.org/talking-about-medications-workshop-materials/) and made freely available for download (Figure 1). The team hosted a public webinar in January 2021 (advertised through Twitter/emails to stakeholders) to share the materials and to explain how other health care providers could host the workshops themselves.

## Assessing the workshop experience

We collected participant demographic information (in a preworkshop survey) and conducted a postworkshop participant satisfaction survey (Appendix 2, available online at www.cpjournal.ca). Field notes were taken by the facilitator and project coordinator during each workshop to document observations, experiences and interactions with participants. Following each workshop, notes were compared and observations and lessons learned discussed. Speaker notes were modified if needed. Data from the January 2021 webinar attendance and workshop material downloads were collected and the project team has since corresponded with people implementing the workshops elsewhere.

### Workshop uptake, satisfaction and our reflections

Seven older adults attended all 3 workshops (age range, 60-78 years); 1 participant only listened during the first workshop and did not join after. Seven participants completed the preworkshop survey, while 6 completed the postworkshop satisfaction survey. Five respondents were female, and all had attained at least a secondary school education.

Box 1 **What are people saying?**

Workshop participants:

• “I found the group that we were with really very willing to participate. And so, the mix of people we were with really made our sessions interesting, valuable and very comfortable.…”

• “One of the things I did find is not what I could do but what I should do now, with my doctor, when I want to talk to her about my medication.”

New workshop facilitators:

• “I am looking forward to the opportunity to utilize your Workshop. Your organization did a great job and a great service in putting this together.” (Pharmacist Consultant, USA)

• “I just want to let you know that I have started offering the first series of ‘Talking About Your Medications’ workshops since attending your training in January. Thank you for putting all the great resources together!” (Ontario Family Health Team pharmacist)

All participants seemed actively engaged throughout each workshop, and their understanding of polypharmacy and deprescribing seemed to grow (e.g., understanding “polypharmacy” as taking many medications vs filling prescriptions at multiple pharmacies or taking different classes of medications). Participants also recounted personal anecdotes of their challenges in keeping track of their medications and shared strategies for improving medication management and for talking about their medications with their providers. By the third workshop, participants shared how they had updated their medication lists, with 1 participant mentioning that they had asked their care provider for additional information about a medication they were taking.

All found the workshops informative and enjoyable and said that they would recommend the series to others. Two participants attended a 1-hour follow-up discussion, held a week after the final workshop. They provided useful feedback on the Participant Workbook, which was combined with feedback received from external reviewers to inform revisions to workshop materials.

### Webinar attendance and uptake of materials

The January 2021 webinar was attended by 85 (mostly) health care providers (pharmacists, physicians, nurses, members of a health team, health researchers) from 8 provinces (British Columbia, Alberta, Saskatchewan, Manitoba, Ontario, New Brunswick, Nova Scotia and Newfoundland) and 6 other countries (USA, Spain, New Zealand, Austria, Australia, Brazil). Following the webinar, attendees were asked about their intention to host the workshops. Of 21 respondents, 95% (20/21) said that older adults in their community would be interested in attending these; 76% (16/21) intended to personally host the workshops. As of November 16, 2021, the workshop Implementation Guide, Facilitator’s Toolkit, Participant Workbook and Workshop slides have been downloaded 56, 68, 58 and 57 (workshop 1), 48 (workshop 2) and 49 (workshop 3) times, respectively. These data support that our interactive educational workshop is being adopted beyond our region, with materials being accessed widely (from Canada, the USA, the UK, Australia, Hungary, India, New Zealand and Brazil).

## Implications—how the workshops can help patients

There were many lessons learned from the implementation of our pilot interactive workshop series, particularly with its virtual delivery format. We summarize these below and share our insights.

### Interactivity in a virtual group discussion format

Ensuring interactivity made participants active contributors to the collective learning of the group. With the facilitator intentionally opting to deliver the workshop content without slides, this gave the workshops a “round-table discussion” atmosphere vs a didactic classroom setting. Anecdotes shared by participants about their own experiences with polypharmacy, medication management and talking with their providers about medications served as important, real-world examples of these concepts. Such examples enabled participants to grasp workshop teachings more quickly. The use of worksheets and homework helped participants to review concepts and apply the strategies taught, with the facilitator readily available to provide guidance as needed.

The virtual group discussion format allowed active participation by learners, asking questions when a topic was unclear and supporting each other in the learning process. For example, during Workshop #2 on medication management strategies, 1 participant was unfamiliar with blister packs as a medication management tool. Another participant retrieved their own blister pack, brought it on-screen to show to the group and explained its use in medication management, without any prompting from the facilitator. With some additional input from the facilitator, participants were left with a better understanding of the tool. The group discussion format permitted many spontaneous learning opportunities such as these.

Given these observations, we recommended workshops include interactivity through use of a group discussion format.

### Workshops as a means to foster social connectedness

An unintended benefit of our workshop series was the social connectedness that developed between participants. Social connectedness has been defined as “a short-term experience of belonging and relatedness, based on quantitative and qualitative social appraisals and relationship salience.”^29^ Throughout the workshops, the facilitator and coordinator endeavoured to create a welcoming and safe environment, where participants could share personal information. During workshops, participants shared their experiences and challenges with polypharmacy, medication management and interactions with health care providers. The workshop content and format permitted participants to be vulnerable and open with each other, allowing them to connect with others’ experiences and provide advice or support. In a time where many older adults have struggled with social isolation and loneliness due to COVID-19 control efforts,^30,31^ our workshops appeared to provide a forum that allowed people to develop meaningful connections with others. As participants primarily formed connections through their shared experiences and challenges with medications, it is conceivable that this can be preserved if workshops are delivered using an in-person discussion format.

### Challenges and opportunities with virtual delivery

Delivering a workshop series using a virtual platform was a new endeavour for our research team. We initially hypothesized that virtual delivery and preservation of the interactive, discussion format of the workshops would be fraught with technical barriers and prevent older adults from freely and fully participating. While we encountered challenges, they were manageable with the assistance of a technical staff member with prior knowledge and training in the virtual platform. By involving this person in the planning process and during the live workshops, we were able to address the technical issues, enabling the facilitator to stay focused on delivering the workshop material. We found it was important to be patient with participants and creatively adapt when technical “hiccups” occurred. For example, 1 participant had a nonfunctional microphone; to elicit their input, the staff member asked that their comments be typed in the chat box, to be read by the facilitator. This enabled the participant to contribute to the discussion and connect with their peers. Another example was when the video modelling a shared decision-making conversation did not display properly over Zoom’s “Share Screen” feature. The staff member quickly uploaded the video to YouTube to be played later, signaling the facilitator to continue delivering the content. To mitigate technical issues that may arise when delivering virtually, we recommend a meeting with each participant ahead of the first workshop to test out the platform.

Undertaking a virtual delivery of the workshops resulted in unintended positive consequences, such as allowing us to connect with a group of seniors during a period of social isolation, not being affected by time of year or weather, enabling those with mobility issues to participate and incurring no cost to book a facility. Virtual delivery can also allow accessibility to participants from a wide range of geographic areas.

## Conclusions

Overall, our experience was positive in developing and delivering this virtual workshop series designed to help older people gain knowledge and skills in managing their own medications and to participate in shared decision-making conversations. The content was of interest to registrants and also to those who downloaded the materials from our website. While initially a rigorous evaluation of learning and self-efficacy in a face-to-face environment was planned, we were unable to complete this due to the limited number of virtual participants. Through our experience, we are pleased to share the workshop materials to enable others to use these to engage with members of the public in supporting their medication management.
